# Reviews and Book Notices

**Published:** 1880-03

**Authors:** 


### Reviews and Book Notices.

ARTICLE X.—A TREATISE ON THE THEORY AND PRACTICE OF MEDICINE. By JOHN SYER BRISTOWE, M.D., London. Fellow and formerly Censor of the Royal College of Physicians; Senior Physician to and Joint Lecturer in Medicine at St. Thomas' Hospital; Examiner on Medicine to the Royal College of Surgeons; formerly Examiner in Medicine to the University of London; and Lecturer on General Pathology and on Physiology at St. Thomas' Hospital. Second American Edition revised by the Author. With Notes and Additions by JAMES W. HUTCHINSON, M.D., one of the Attending Physicians to the Pennsylvania Hospital; Physician to the Children's Hospital, Philadelphia. Philadelphia: Henry C. Lea, 1879.

This is a full-sized octavo volume of 1,081 neatly printed pages, with good leather binding. It is a general work on the theory and practice of medicine, written expressly for a text-book for the use of students. As this is the second American edition, it would be pleasant to join most of our cotemporaries in simply saying that the work is already too well and favorably known to the profession to require an extended notice at our hands. But having recently, in a brief notice, found some fault with the new volume on Clinical Medicine, by Dr. A. Flint, we could not consistently pass the present work with a mere general commendatory remark.

The work before us is divided into two parts. Part I, General Pathology, occupies the first one hundred pages of reading matter, and includes a brief discussion of the Definition of Disease; the Etiology of Disease; Physiological Processes in Health; Physiological Process in Disease, and the Treatment of Disease.

In regard to the first topic here mentioned, the author, after three pages of comments and illustrations, states his definition of disease as follows :

" And hence, if these views be generally true, disease may be defined *as a complex of some deleterious agency acting on the body, and of the phenomena (actual or potential) due to the operation of that agency.*" (Page 36.)

The italics are the author's, and the words thus emphasized clearly show that he includes in his idea, as expressed by the word *disease*, three distinct things, namely, the cause or causes ; the morbid conditions or disease proper ; and the phenomena or symptoms by which the latter is manifested. When the writers of two or three generations past, defined disease by enumerating a group of symptoms, and designated the morbid or pathological condition as the *proximate* cause ; and the causes or agencies inducing such morbid condition or proximate cause, as predisposing and exciting causes, they preserved, at least, a logical clearness of statement. They misplaced the word disease, by using it to designate the symptoms or phenomena of morbid conditions, instead of the conditions themselves ; but they avoided confounding together things or ideas essentially distinct. Our present author, however, insists on making confusion worse confounded in the mind of the student, by including in the meaning of one word, disease, causes, morbid conditions, and consequences or symptoms. That I do the author no injustice here, I will quote his own illustration directly following his definition of disease as already given, from page thirty-six.

He says : "All diseases involve, some in a greater, some in a lesser degree, certain groups of pathological consequences immediately traceable to their respective morbid causes ; but these primary pathological consequences themselves tend to evoke others, these again a tertiary series, and so on continuously.

" Thus a person with carcinoma of the bowel may, as a consequence, have stricture, or perforation, or involvement of the glands occupying the retro-peritoneal tissue and gastro-hepatic omentum, or that form of cachexia which cancerous disease so frequently induces ; and, as a consequence of these several secondary morbid conditions, various other affections, such as enteritis, peritonitis, jaundice, ascites, melæna, thrombosis or anasarca. Now all these phenomena, and many others, are obviously integral portions of the carcinomatous affection which the patient is suffering, and all of them may be regarded as symptoms or incidents of that affection ; but many of them are not unfrequently also looked upon as quasi-independent disease, and treated as such. There is no doubt that they are *not* diseases. They are clearly however, elements of disease ; and inasmuch as each one of them arises out of some immediately antecedent abnormal condition which is its direct cause, they do obviously enough, in association with their respective causes, fall severally within our definition of disease."

Here the author apparently for the sole purpose of making it appear that a definition of disease should embrace, not only the disease proper, but also its causes and consequences, allows himself to call such distinct morbid conditions as stricture and perforation of the intestine, enteritis, peritonitis, etc., mere "phenomena," "symptoms or incidents" of a prior disease ; "*integral* portions of the carcinomatous affection ;" and to declare "that they are not diseases," but "elements of disease." If any one can explain how a morbid condition like peritonitis, for example, can be at one and the same time, an "element" or "integral portion" of a prior carcinoma of the bowel, and a "phenomenon," "symptom or incident" of such disease, he will certainly exhibit a rare degree of skill in reconciling incongruities in the use of language. Is a peritonitis, enteritis, or stricture any the less a disease, because it exists as a complication of carcinoma of the bowel, which may have acted either as a predisposing or exciting cause ?

Certainly not. The author carries the same confusion of ideas, or inaccuracies of expression into his general comments on etiology. Instead of dividing the causes of disease into simple predisposing and exciting, he follows the example of earlier writers and divides them into predisposing, exciting, and proximate ; and gives the usual definition of each class, declaring the *third* class to comprise "those morbid processes which the action of the exciting cause calls into play, and to which the symptoms are supposed to be directly due."

Yet he immediately adds: "The proximate cause indeed, is often, though *erroneously* said to be the disease itself." But why erroneously? If, as he has just asserted, the *proximate* cause comprises "those morbid processes," or deviations from the healthy standard of action, "which the action of the *exciting* causes calls into play," what is it but the disease itself?

If the morbid processes or changes set up in a living structure or organ by action of some exciting cause, do not constitute a disease, pray what does? Our author, however, proceeds to illustrate as follows (See p. 37):

"A woman, who has frequently been exposed to the contagion of scarlet fever without taking the disease, is again exposed at the period of child-birth, and now suffers from a virulent attack. Here, peritonitis, which renders woman peculiarly susceptible of the contagious fevers, is the *predisposing* cause; the scarlatinal contagion is the *exciting* cause; and the inflammatory processes going on in the skin, tonsils, and elsewhere, the *proximate* causes of most of the symptoms which the patient manifests."

So far all is clear, "But," he immediately adds, "the *exciting* cause of the scarlet fever is obviously the *proximate* cause of that disease, and the *proximate* causes of its several secondary phenomena are just as obviously their *exciting* causes." He had just stated that the exciting cause was the scarlatinal contagion or poison, and that the inflammatory processes going on in the skin, tonsils, etc, were the proximate cause. Now he declares the exciting cause of the fever to be the proximate cause. In other words, the cause of the disease is the disease itself. The student who is expected to acquire logical modes of thought, and clear conceptions of the relations between causes and effects, from the study of the first part of Dr. Bristowe's work is not to be envied.

The great body of the work is occupied by Part II., Special Pathology, or the consideration of individual diseases.

These are arranged under eight heads or chapters as follows: 1. Specific Febrile Diseases; 2. Diseases of the Skin; 3. Diseases of the Respiratory Organs; 4. Diseases of the Vascular Organs; 5. Diseases of the Digestive Organs; 6. Diseases of the Genito-Urinary Organs; 7. Diseases of the Organs of Locomotion; 8. Diseases of the Nervous System.

The details of this part of the work are full and comprehensive, so far as the number of subjects treated, is concerned. And though we should be obliged to differ with the author in regard to some etiological and pathological doctrines, yet as a general rule his account of the symptoms, progress, and diagnosis of diseases is concise, clearly stated, and satisfactory.

Sometimes, however, his descriptions are lacking in accuracy, and he not unfrequently uses words of specifically different meaning as promiscuously as though they were synonyms. But the least satisfactory part of the whole work, so far, at least, as it is used by students and inexperienced practitioners, is that relating to the treatment of individual diseases. In most cases his views concerning the remedial treatment of diseases, are expressed in the most brief and general terms, with no adequate adjustment of the remedies to the different stages in the progress of the morbid processes, nor any allusion to the variation of doses required to accomplish given results, in different morbid conditions. Take as example, variola or small pox. After occupying seven closely printed pages in describing the causes, symptoms, progress, diagnosis, etc., his entire account of the internal treatment of the disease is embraced in the following quotation, occupying about one-third of page 174:

" *Treatment.*—In the mildest forms of small-pox medicinal treatment is scarcely called for; in the severest it is useless; and indeed, under any circumstances, it has but little influence over the course of the disease. The patient should be placed in an airy chamber, which should be well ventilated, and kept at a uniform and medium temperature. He may take as medicine some cooling drink—lemonade, soda-water or other saline or acidulated solution. If the bowels be confined, they may be acted upon by some mild laxative; if there be diarrhœa (especially in adults), they must be restrained by opiates, or other astringents. The soreness of the throat may be relieved by warm bland drinks, or black-currant jelly; and, if there be much discharge from the nose and about the fauces, these parts may be washed with some mild detergent or astringent solution.

Opium is often of value both in relieving the delirium and assuaging the pain of the invasion period; but it is especially useful during the period of secondary fever. If there be great tendency to collapse, ammonia may be serviceable. Nourishment should be regularly administered, and should consist of the materials generally suitable for febrile conditions, namely, milk, rice-water, gruel, beef-tea and such like. Alcoholic stimulants must be given according to circumstances; but are *especially important* in the malignant form of the disease, and in the later periods of confluent, small-pox, or whenever there is tendency to collapse."

Here, we are told in the first paragraph, that all medicinal treatment is either unnecessary or useless; and in the last, that certain remedies are "especially important," in the very same class of cases that they are declared to be "useless" in the first. We are told that, "if there be great tendency to collapse, ammonia may be serviceable," but which of the half dozen preparations of ammonia is to be used, or in what doses, the student is left to guess. The entire treatment of yellow fever occupies about one-third of a page; that of enteric or typhoid fever less than two pages; and that of pneumonia nearly one page. In regard to this last-mentioned disease, the author makes no allusion to the very important modifying influences of coincident typhoid and malarial agencies, either upon the progress or treatment of the disease: yet every practitioner of experience, in this country, can testify to the very great importance of giving due heed to the influence of these agencies in the remedial management of this grave and frequently-occurring disease.

It is not necessary to extend the comments upon this work further, or to particularize further defects. It is sufficient to add that it is not such a text-book on the theory and practice of medicine as is most desirable for the use of students and young practitioners. N. S. D.

